# Possible Mechanisms for the Effects of Sound Vibration on Human Health

**DOI:** 10.3390/healthcare9050597

**Published:** 2021-05-18

**Authors:** Lee Bartel, Abdullah Mosabbir

**Affiliations:** 1 Faculty of Music, University of Toronto, Toronto, ON M5S2C5, Canada; amosabbir@research.baycrest.org; 2 Baycrest Health Sciences Centre, Rotman Research Institute, Toronto, ON M6A2E1, Canada

**Keywords:** vibration, whole body vibration, vibroacoustic therapy, physioacoustic, hemodynamic, neurological, musculoskeletal, music as vibration, music and health

## Abstract

This paper presents a narrative review of research literature to “map the landscape” of the mechanisms of the effect of sound vibration on humans including the physiological, neurological, and biochemical. It begins by narrowing music to sound and sound to vibration. The focus is on low frequency sound (up to 250 Hz) including infrasound (1–16 Hz). Types of application are described and include whole body vibration, vibroacoustics, and focal applications of vibration. Literature on mechanisms of response to vibration is categorized into hemodynamic, neurological, and musculoskeletal. Basic mechanisms of hemodynamic effects including stimulation of endothelial cells and vibropercussion; of neurological effects including protein kinases activation, nerve stimulation with a specific look at vibratory analgesia, and oscillatory coherence; of musculoskeletal effects including muscle stretch reflex, bone cell progenitor fate, vibration effects on bone ossification and resorption, and anabolic effects on spine and intervertebral discs. In every category research on clinical applications are described. The conclusion points to the complexity of the field of vibrational medicine and calls for specific comparative research on type of vibration delivery, amount of body or surface being stimulated, effect of specific frequencies and intensities to specific mechanisms, and to greater interdisciplinary cooperation and focus.

## 1. Introduction

Music has the power to affect our health and wellbeing. This belief and related cultural practice has been evident throughout history [1,2], but the clinical profession of music therapy is only some 75 years old. As a field of practice music therapy has been described as an art and a science and regards to science its research foundation has drawn on psychoanalytic, humanistic, and behavioral traditions [3]. As an emergent practice within the healthcare context, the focus of music therapy (a therapeutic relationship employing music as a means) and music medicine (the music or sound itself as the therapeutic) research has been primarily evidence-based. Mechanisms of effect have received little attention until recently. To develop clinical and scientific legitimacy in general healthcare and expand its use as standard of care, research on the use of music must show both that it works (evidence-based research) and why it works (mechanism-based research).

Part of the problem of creating a scientific foundation for music effects in healthcare is that music is a very complex phenomenon; to control its parameters scientifically is practically impossible. That is, if music is approached as “music”—a cognitively constructed product of a culturally situated acoustic phenomena—the variables that can affect a response are practically limitless. Efforts to provide more detailed reporting of the music used in research are a start [4]. However, another approach is to define the potential level of the response mechanism.

### 1.1. Basic Concepts

#### 1.1.1. Mechanism

Focusing on the mechanism itself can be highly complex. Craver and Bechtel [5] describe four dimensions of mechanisms: (1) a phenomenal dimension—a mechanism does things. For music the human ear offers a basic mechanism illustration. The ear translates compression and decompression of air molecules into the conscious perception of sound. (2) A componential dimension—a mechanism has components. In a simplified description the ear has components including the ear drum, the malleus, incus, stapes, and cochlea that transmit the vibration to the cilia which translate the physical vibration to an electrical signal for the auditory nerve. (3) A causal dimension—components interact to make the phenomenon happen. The components of the ear turn the air compressions into electrical signals transmitted through the auditory nerve to the internal perceptual processing system (a complex mechanism in itself). (4) An organizational dimension—components are organized in space and time. There is a particular organization of the components of the ear and how they interact that causes vibration to be perceived as sound.

The basic question of what are the mechanisms of how people enjoy music illustrates a very important aspect of the challenge of studying mechanisms—the ear is essential in the perception of music but it is only a start to the understanding of the cascade of mechanisms or causes of music enjoyment. That understanding would involve further definition of the auditory nuclei in the brainstem, the role and function of the thalamus, the means of transmission to the auditory cortex, the role of memory in the perceptual process, the interaction of expectation with neurotransmitters like dopamine and their role in the perceived response, and so on. Similarly, the question of how does low frequency sound stimulates blood circulation leads to an examination of endothelial cell response resulting in nitric oxide release and its effect on blood flow and so on.

The crucial point here, and an important caveat for this paper, is that research into the mechanisms of the effect of music on health is nascent and in most cases focused on the most obvious components of what can be presumed to be a complex system [6,7]. This paper will, therefore, attempt to point in the direction of potential mechanisms that appear to be emerging for further study rather than serve as a detailed explication of a set of defined mechanisms.

#### 1.1.2. Types of Stimulus and Response

A starting point to study mechanisms is to try to categorize by type of stimulus and type of response. One of the difficult pivots in the thinking of many health researchers is to differentiate between “music” and “sound vibration” as the stimulus. The bulk of research by cognitive neuroscientists focuses on “music” and often with little control of the parameters of that music since the assumption is that the effect stems only from the cognitive processing of that music [8,9].

Within the broad category of “music” as stimulus, the response focus can be on the general effects like arousal, mood, emotion, relaxation, stress, etc., and with the assumption that these responses result from enjoyment or aversion reactions, memory associations, or some inherent neurological process [8]. All of these responses can be attributed to learned schemas, processes, and associations developed through socially mediated cultural immersion from the very earliest days of exposure to music [10].

A second category of mechanisms within “music” is to look at the effect of music cognition on the activation of neural circuits and specific neural functions. This has been most clearly defined in relation to rehabilitation [11,12].

When music or audible sound is considered for its vibratory (rhythmicity) effects, a third category of response mechanism emerges and its focus becomes primarily cellular [10]. The most basic and oldest use of vibratory effect is the mechanical “shaking” of the body and more recently specific areas and cells. Another more specific category of cellular response is the effect of driving a neural modulatory response. The importance of rhythmic brain activity, and the potential for sensory stimulation to drive it, has not received much attention until recently [13,14]. However, for music as a vibratory phenomenon this is an important mechanism to study.

#### 1.1.3. Vibratory Rhythmicity

Fundamental to vibratory effects is the rhythmicity of music. It can be thought of as events per second and seconds per event [15]. When there are more than 16 events per second cognitive event fusion changes from individual event perception to hearing pitch frequencies. The rhythmic stimulation effect on cells, however, continues to be related to the events per second even, for example, at 40 or 60 events per second (Hz). If the events are slower than about 10 s per event, long-term memory may allow perception as functional units of musical form but these will not be heard as individual events. Body rhythm cycles, like the 10 s baroreceptor control loop, may still be response to entrainment by recurring events at this frequency and influence these Mayer waves to release endothelial derived nitric oxide [16]. Between 16 events per second and 10 s per event lies the area where short-term memory allows cognitive perception of the individual events as notes, patterns, or melodic groupings. In this category people align physical movement like running, walking, dancing, foot or finger tapping, or mental tracking with the beat. This category of cognitively tracked rhythmicity has known effects of neural, cardiac, and breath entrainment and is used therapeutically, for example, in rhythmic auditory stimulation (RAS) [11]. However, it also has direct effects on cells through mechanical movement.

This paper will not focus on music as “music”; it will focus exclusively on the rhythmicity of music and sound as vibration. In many cases the focus is on the effect of single frequencies although these frequencies may be imbedded in music and heard by the recipient simply as music.

### 1.2. Vibratory Applications to Health

#### 1.2.1. Historic Predicates

It is common to encounter studies of the effect of vibration and low frequency sound in industrial-oriented research. These studies typically focus on the potentially negative effects of specific work environments, use of particular equipment, and primarily exposure to low frequency vibration and noise [17,18]. However, there is also a long history of interest in the positive effects of vibration. Carriage rides on rough cobblestones came to be associated with positive health effects and mechanical “chairs” emulating these rides were created. In the 18th century the Abbéde St. Pierre created the tré moussoir or fauteuil de poste as a vibrating chair to help patients with melancholia, liver disease, and other conditions that seemed to respond to carriage rides [19]. The 19th century saw considerable interest in vibration and the development of vibrating tools including the chair created by Dr. Jégu after seeing Parkinson’s disease (PD) symptoms calmed by a carriage ride [19,20]. Dr. Jean-Martin Charcot, the most noted neurologist of that century, began studying PD with the chair but acknowledged an earlier physician, M. Vigoroux, who in 1878 used a sounding box with a very large attached tuning fork played with a bow to successfully treat patients with hemianesthesia and locomotor ataxia [21]. Charcot also described treatment of neuraglia and migraines by Dr. Boudet of Paris with tuning forks mounted on small boards. Gilles de la Tourette, Charcot’s assistant, applied the idea of vibration therapy to the brain [19,20,21] with a metal helmet he devised with a motor on top causing the helmet to vibrate at 10Hz. It was found to have a positive effect on insomnia, migraines, and depression and other vague conditions [21].

#### 1.2.2. Current Therapeutic Application Concepts for Vibration

Whole body vibration (WBV), also referred to as whole body periodic acceleration (WBPA) is one type of application that grew out of the 18th and 19th century interest in vibration. WBV, a mechanical vibration typically created with stand-on oscillating platforms, developed largely in response to concerns about the effect of weightlessness in space on bone and muscle and then was quickly applied in sports [22]. Although current WBV platforms can produce vibration frequencies up to 100 Hz, frequencies below 30 Hz are most commonly used. The past 20 years has seen growing interest in the effects of WBV on bone density, orthopaedic, and neurological concerns [23,24].

More in keeping with the early uses of tuning forks for sensory stimulation is the practice of low frequency sound therapy (and most closely related to music therapy) that has developed in the past 50 years and is now commonly known as vibroacoustic therapy (VAT). WBV typically uses frequencies below 30 Hz while VAT uses 30–120 Hz. Olav Skille in Norway and Petri Lehikoinen in Finland were the leaders in this use of sinusoidal sound to stimulate the body for therapeutic purposes. Skille placed particular emphasis on single pitches at 40, 52, 68, and 86 Hz modulated with a steady rise and fall of amplitude at a rate of about 6.8 s from peak to peak. A noteworthy application of this method in research was done by Wigram [25,26]. Instead of single frequencies, Lehikoinen used constant frequency scanning with the intent to treat muscles at their particular resonance frequency, slow power pulsation to prevent muscle contraction, and at times directional movement of the sound [27]. Lehikoinen developed the NextWave chair system that was Federal Drug Administration (FDA) and British Standards Institution (BSI) approved in 1996 for three claims related to physioacoustic therapy: increased blood and lymphatic circulation, decreased pain and stress, and increased muscle relaxation and mobility [28]. Numerous devices that include chairs, chair backs, beds, mats, pillows, backpacks, and smaller hand-held units have been developed since then.

### 1.3. Definitions, Clarifications, and Terminology

#### 1.3.1. Source of Pulsed Stimulation: Sound Waves or Mechanical Compression

To review the literature related to vibration, one of the fundamental questions requiring clarification is whether the stimulation of the body with sound waves is different in some way from mechanical vibration. Since people hear sound waves and feel mechanical vibrations, an easy conclusion is that the two are categorically different. However, sound in essence is mechanical vibration that transmits through a medium [29]. In the medium of air, the sound actuator creates a vibration that results in regular compressions and decompressions of air molecules that travel to the receiving surface on the body such as the ear’s tympanic membrane or the mechanoreceptors in the skin. In water a rapidly oscillating membrane would create compressions and decompressions of water molecules. Once in contact with the body, the compression and decompression of the surface of the body is transmitted through bone and tissue and may be sensed by a set of mechanoreceptors or by our auditory system.

At a cellular or molecular level in the body there is probably no difference between activation by air molecules applying regular sine wave pressure on the body, by a surface applying oscillatory pressure stemming from a rotating motor shaft, or by the body itself moving against gravity on an oscillating platform. Another way to understand this can be in comparing the application of sound to produce vibration and the application of vibration to produce sound. Sound, which propagates through a material can vibrate the material and be physically felt like a massage, such as in a vibroacoustic device [30]. Mechanical vibration can also produce sound. Bone conduction headphones are commercially available and are built to be positioned along the skull. The mechanical vibration of the bones of the skull propagate to the inner ear and are perceived as sound [31]. Therefore, the interchange of sound and mechanical vibration demonstrate that they are in essence the same thing.

#### 1.3.2. Vocabulary and Inclusion

Clarification is needed about the vocabulary used in this field. We have already explained VAT and WBV. Another term used is rhythmic sensory stimulation (RSS) and is inclusive of multiple types of pulsed (rhythmic) stimulation. RSS includes whole body rhythmic movement, vibrotactile stimulation of all or part of the body, auditory pulses delivered as individual sound units (like hits on a drum, plucks of a string, interaction “waves” resulting from binaural detunement, or isochronous amplitude modulated sound trains) or as molecular compressions that create continuous sound (research usually focusing on low frequency sound 20–130 Hz), and visual light flashes or flicker. Pulsed ultrasound can also be regarded as a type of vibrational mechanical stimulation and is typically applied in 2 ms bursts but with varying ratios of sound to silence from 1:1 (250 Hz) to 1:20 (45 Hz).

When considering the use of rhythmic pulses to stimulate to the body, one question is whether electric stimulation and pulsed ultrasound are comparable to mechanical vibration. Bartel et al. [32] drew parallels between pulsation frequencies used in electric stimulation and pulsed ultrasound with frequencies for VAT. Many years ago Charcot postulated that the vibration stimulation was not unlike electric stimulation and showed similar results [21]. In this paper the review of specific mechanisms activated by vibration will be restricted to studies using various sonic and mechanical vibratory means from general body vibration to focused points of vibration delivered with pencil-like probes. However, a future more extensive review of the mechanisms activated by pulsed stimulation might include electrical and pulsed ultrasound stimulation as well.

A final question is whether whole body vibration (WBV) is fundamentally different from vibroacoustic therapy (VAT) and, therefore, WBV studies cannot be mixed with VAT in the examination of mechanisms. First, although many applications of WBV use frequencies in the level of infrasound (1–15 Hz) they also use higher frequencies (e.g., 20–40 Hz) in the same range that VAT frequently uses. Music stimulation and VAT can employ pulsed sound units at 1–15 Hz and, in fact, “rhythm” in music is primarily in that frequency. So, there is no set frequency that makes it one or the other. Secondly, the axis of the applied vibration may be a discriminator between approaches (e.g., vertical (axial), horizontal, or multidirectional) but at the mechanism level there does not appear to be strict differentiation. For example, with blood flow slow axial (direction of the spine) WBV creates pulsatile stress on endothelial cells and so enhances blood flow at 1 or 2 Hz [33] but also with sound stimulation at 50 Hz [34]. Rhythmic driving of oscillatory coherence happens with rhythmic pulses at 1 or 2 Hz (delta entrainment, e.g., RAS effects) and at 10 Hz (alpha), 20 Hz beta, or 40 Hz gamma, etc. Consequently, at the level of the mechanism, WBV and VAT will be considered within the same domain of pulsed stimulation.

### 1.4. Methodology and Scope

#### 1.4.1. Narrative Review Approach

In the next section we will present a narrative review of research literature that tries to map the basic “lay of the land” of vibration stimulated mechanisms and what health conditions may be affected. This is not a systematic critical review of research methodology, but rather a “cataloguing” effort to describe applications of vibration and mechanisms of effect. This effort was motivated by the perception that this has not been done to date. Researchers working on the mechanisms of vibration effect have essentially worked in silos. We determined to look for commonalities among the silos by looking not at the vibratory device, frequency, or context of application, but rather at mechanisms involved. Consequently, our search for studies to include was an emergent process from general to specific with the intent to identify categories of mechanism and then the specifics within the categories. We used a basic strategy to locate candidate studies: an electronic database search including PubMed, Scopus, and Google Scholar. Initial searches included terms such as “Vibration and Health”, “Vibration mechanism”, and “Vibration treatments” from years 1975 to 2021. We filtered based on number of citations and considered the more cited work first. After an initial collection of literature and, as researchers starting with the greatest awareness of VAT where the 1996 FDA claims for blood circulation, pain reduction (neurological), and mobility (musculoskeletal) effects were established, we decided to focus on three central concepts of vibration mechanisms for further searches: the effects of vibration on the blood, the nerves/brain, and bone/muscle. A further refined search included terms like “vibration and blood mechanism”, “vibration and neuron mechanism”, “vibration and brain”, “vibration and bone/muscle mechanism”, etc., followed by specific mechanism like endothelial stimulation or mechanoreceptor response. The identification and selection of papers was driven primarily by an attempt to describe the “field”, the “lay of the land” of vibration research. Further, our focus was closely held to mechanism and not evidence-based clinical research related to vibration without attention to mechanism. Papers were chosen based on whether we believed them to add to our understanding of the effects of vibration on health via those areas.

#### 1.4.2. Mechanisms of Response to Vibration

The auditory and vibrotactile stimulation from low frequency sound shows effects that are essentially the result of two categories of mechanisms: (1) physical, through muscular and cellular means, and (2) neurological, through sensory-based stimulation of nerves and receptors. At the physical level sound vibration is sensed by tactile receptors in the outer skin (Merkel disks—sensing vibratory strength and responding most to 5–15 Hz), inner skin (Meisner corpuscles—sensing vibratory frequency and responding most to 20–50 Hz), and in deeper tissues (Pacinian corpuscles—sensing acceleration and responding most to 60–400 Hz) [35,36]. To avoid numbing of these sensors, VAT is usually constantly varied in amplitude (power pulsation) and/or frequency (scanning). A physical therapeutic effect can be obtained at a cellular and lymphatic level due to increased fluid and cellular waste transport, increased cellular metabolism [37,38], increased blood circulation, and muscular relaxation due to a resonance response. Within the brain, vibration hypothetically enhances flow of cerebrospinal fluid and speeds removal of metabolic waste [39]. Most research with VAT has not explored neural oscillatory effects but recent studies show [40,41,42,43,44] potential brain effects, especially through prolonged application of a single frequency (e.g., 40 Hz).

In 1996 the Federal Drug Administration (FDA) reviewed the Next Wave Physioacoustic Chair designed for medical purposes with six imbedded subwoofer speakers used to apply low frequency sound vibration to the body from knees to shoulders at frequencies from 20 to 130 Hz. The FDA approved three claims for the treatment at the time called physioacoustic therapy [28]: (1) increased blood circulation, (2) decreased pain, and (3) increased mobility. Although no evidence was provided at the time for mechanisms of action, the three areas generally pointed to hemodynamic effects, to neurological effects, and to musculoskeletal effects. This FDA approval is important and noteworthy because it appears to be the earliest vibration device approved for multiple claims. Although whole body vibration (WBV) devices have been used for many years with the intent of developing bone and muscle strength, FDA approval was not granted [45]. More recently specific limited application devices using vibration have been cleared for sale [46,47]. However, the effects claimed for WBV and the recent devices fit into the original categories of claim from 1996. Further the general broad categories for the claims for physioacoustic therapy encompass the vast majority of mechanisms we discovered in the literature. The exceptions were primarily in the effects of mechanical shaking related to phlegm and mucous [48] or orthodontics [49].

### 1.5. Organization of the Analysis of Mechanisms

In the following three sections we explicate three primary categories of mechanisms activated by vibration applied to the body: hemodynamic, neurological, and musculoskeletal. By using the concept of mechanism established by Craver and Bechtel [5] we consider the causal and organizational dimensions to identify what we call basic mechanisms and submechanisms. This is a utilitarian simplification to focus, what is in most cases a complex system, on the organizational sequence. For example, vibration results in stimulation of endothelial cells (basic or first step mechanism), which then in turn releases nitric oxide and adrenomedullin, which have different effects and so are designated submechanisms. As we have stated earlier, this will not be an exhaustive and definitive description of complex chains of mechanistic actions. Rather we are setting out to point to directions for further research investigations of the effects of vibration on the body. In addition to identifying basic causal mechanisms, we describe some health related applications. Not every mechanism described in research has clear clinical applications. This is a nascent field where much more research is required and where knowledge translation into clinical application is needed.

## 2. Hemodynamic Effects

### 2.1. Basic Mechanism: Stimulation of Endothelial Cells

#### 2.1.1. Submechanism: Nitric Oxide

One of the effects of vibration applied to the body acknowledged by the FDA in 1996 was an increase in blood circulation [28]. The question is by what mechanism does vibration affect blood flow. A crucial signaling molecule that has a pivotal role in regulating blood flow and oxygenation of tissues is nitric oxide (NO). Nitric oxide is produced and released into the blood by the endothelial cells that line the blood vessels and lymphatic vessels. Vibration stimulates the endothelial cells to produce and release NO in several forms of which the isoform endothelial nitric oxide synthase (eNOS) is of particular importance for the generation of NO from endothelial cells. Although the detailed mechanisms of how sound affects the mechanosensors in the endothelium to control eNOS is not yet completely understood, it appears that the endothelial cells mechanosensor-proteins, Syndecan-4 (Syn4) vascular endothelial growth factor (VEGF), and Krüppel-like Factor 2 (KLF2) translate the physical force from the vibration into biochemical signals. Studies have shown that vibroacoustic stimulation can induce Syn4 and VEGF [50,51]. NO regulates blood flow and vascular tone by affecting the vascular smooth muscle with the activation of the enzyme guanylate cyclase (sGC) [52] and the phosphorylation of extracellular signal-regulated kinase (ERK1/2) [53]. Although the purpose of stimulation, mode of vibration delivery, and frequency of vibration varies, endothelial cell stimulation releases NO and induces blood flow. The effect has been observed with whole body periodic acceleration using a platform (pGz) at 2 Hz [54], with microcirculation in the skin at 47 Hz [55], externally applied vibration to the arm at 50 Hz [34], sonic vibration applied to the chest at 100 Hz [56], and at various body surfaces at 150–250 Hz [57].

##### Application: General Blood Flow

Since vibrational stimulation induces blood flow, this may find general application to conditions resulting in decreased blood flow like diabetes. Maloney-Hinds [34] used 50 Hz vibrational stimuli for five minutes on participants’ forearms and found NO production increased by 374% in the healthy controls and by 236% in the diabetes group. Skin blood flow was significantly increased (*p* = 0.0001) in both groups. Johnson et al. [58] studied diabetes patients with whole body vibration at 26 Hz and also found significant increase (*p* = 0.01) in skin blood flow but with less effect than Maloney-Hinds using direct to skin vibration.

##### Application: Cardio Protection and Therapy

An approach to stimulation of endothelial cells is “periodic acceleration” (pGz) consisting of repetitively moving the body in a head-to-foot direction at a typical rate between 1–3 Hz resulting in an increase in “pulsatile shear stress” on the endothelium—the friction of the blood against the endothelial lining of blood vessels resulting from the “pulse” of the movement and the gravitational force on the blood. This “shear stress” of the blood against the lining stimulates the release of NO and other endothelial-derived cytoprotective factors.

One application of pGz is as a form of cardiopulmonary resuscitation (CPR) where the body is moved head to foot at a rate similar to standard compression CPR. Adams et al. [59], in an animal study, found pGz as effective as standard CPR in terms of resuscitation survival rate and other acute outcomes but with fewer rib fractures.

An animal study by Adams et al. [60] applied pGz at 3 Hz for 30 min after induced cardiac arrest and resuscitation. The animals receiving the pGz treatment had a smaller reduction in the function of heart contraction (myocardial stunning), greater flow of blood to the heart, kidneys, stomach, and brain, showed less of an increase in Troponin I, a protein released into the blood stream indicating damage to the heart muscles, and a greater increase in eNOS.

The previous two studies by Adams et al. [61] used endothelial stimulation as a post-cardiac arrest treatment. Does it also offer preventative protection? They examined this by studying the occurrence of tissue damage caused by a return of oxygen rich blood following a period of oxygen deprivation (ischemia-reperfusion injury) caused by ventricular fibrillation (VF). Animal subjects were given one hour of pGz stimulation at 3 Hz prior to the induced VF. The animals receiving the stimulation had fewer arrhythmias of the blood circulation pulse, less myocardial stunning, stronger regional blood flow to organs of the body, and less indication of heart damage (Troponin I). Tests showed that the animals receiving the pulsed stimulation at 3 Hz had higher levels of endothelial nitric oxide with the conclusion that elevation of eNOS may have a cardioprotective function.

The studies by Adams reported above focus on heart function and effects where blood volume is constant. Bassuk et al. [62] looked at the effect of one hour of pulsed stimulation (pGz at 3 Hz) before and during significant blood loss from hemorrhage. The results showed significantly less oxygen consumption in the treatment group at all points of the hemorrhage and, after 40 mL/kg of blood loss, better preservation of regional blood flow to stomach, ileum, kidneys, heart, and brain. The authors attribute this effect to the shear stress produced by the motion on the endothelium and the release of nitric oxide.

##### Application: Neuroprotection and Neurotherapy

Aerobic exercise and the pulsed stimulation of whole body periodic acceleration (WBPA), also known as pGz, both have been shown to have positive cardiovascular effects through activation of nitric oxide (NO). In addition, the NO effect is known to induce the brain derived neurotrophic factor (BDNF) and glial derived neurotrophic factor (GDNF) thereby contributing a neuroprotective (prior to damaging events) and neurotherapeutic effect (following damaging events). A study by Adams et al. [33] showed that aerobic exercise significantly increases blood flow to the skeletal and heart muscles but not to the brain while WBPA increase blood flow with BDNF and GDNF to the brain and the heart but not to the muscles. They concluded that rhythmically pulsed stimulation of the endothelium can be a non-invasive strategy for neuroprotection and neurotherapy.

In a more detailed examination of the effects of WBPA on the brains of mdx mice, Lopez et al. [63] found an overload of calcium and sodium ions and an overproduction of reactive oxygen species (ROS) in the neurons resulting in cognitive dysfunction. After WBPA for one hour per day for eight days at 8 Hz, results showed that the elevation of calcium and sodium ions and the overproduction of ROS had been mollified along with an increase in enzymes that protect cells. This study again demonstrated the efficacy of pulsed stimulation of the endothelium to release nitric oxide and a cascade of factors that result in neuroprotection and neurotherapy.

#### 2.1.2. Submechanism: Adrenomedullin

In addition to the release of nitric oxide, the vibratory stimulation of endothelial cells has been shown to release the cell protecting mediator, adrenomedullin (AM). It may function as a hormone in controlling circulation and vasodilation but also serves to stimulate angiogenesis—the growth of new blood vessels—and combats oxidative stress in cells. In this way AM can have a positive effect in cardiovascular disease including hypertension, myocardial infarction, and chronic obstructive pulmonary disease. However, in its function to extend blood supply in cells it may be a negative factor in relation to cancer. Martínez et al. [64] conducted an animal study to examine the effect of one hour of pGz stimulation on AM and found that immediately after the stimulation blood pressure was significantly reduced (from 115 ± 10 to 90 ± 8) and AM level was significantly increased and remained so for 3 h (from 776 ± 176 pg/mL baseline to 1584 ± 160 pg/mL, *p* < 0.01).

#### 2.1.3. Submechanism: Antioxidants

Many disease conditions are linked to oxidative stress. These include cancer, Alzheimer’s and Parkinson’s disease, diabetes, and cardiovascular conditions like high blood pressure, atherosclerosis, and stroke. Release of nitric oxide into circulation is known to have some antioxidant properties. Uryash et al. [65], in a study with mice with high oxidative stress, specifically looked for the effect of pGz on eNOS and antioxidants. The subjects were given pGz for an hour a day and tissue was tested after one, two, and four weeks. The pulsed stimulation resulted in significant expression of antioxidants including glutathioneperoxidase-1 (GPX-1), catalase (CAT), superoxide, and superoxide dismutase 1 (SOD1). It also decreased reactive oxygen species (ROS).

### 2.2. Basic Mechanism: Vibropercussion

One of the features of vibration is that it results in one material striking against another—whether molecule to molecule or cell to cell or bone to bone, etc. The essentially mechanical action can produce health-related effects.

#### Application: Blood Clot Dissolution

To study the effect of mechanically pulsating a blood clot Hoffman and Gill [66] conducted an in-vitro study (outside the body) placing a one-hour-old blood clot into a solution of the medication Heparin typically used as a blood thinner to treat heart attacks or angina. The vessel holding the blood clot was then vibrated at 50 Hz for 20 min with a very low amplitude to simulate the amount of vibration that might be transferred into the body from a surface vibration. The result showed significantly better clot dissolution with vibration (25%) versus no vibration (5%). Hoffman and Gill [67] then basically replicated the study but applying the vibration externally through a 4 cm slab of meat as simulation of human body structure and varying the vibration to 50 Hz applied in short bursts of one per second. In addition to Heparin, they did a sample with Streptokinase, a medication specifically designed to break down clots. They again found the vibration very significantly increased clot dissolution, and with greater effect in the Streptokinase group attributed to enhanced mixing and penetration (or in a sense “drug delivery”) of the lytic agent, which was originally disposed from the clot.

One context where vibration is associated with stroke treatment is the “drip and ship” situation where a patient is started on a drip of recombinant tissue-type plasminogen activator (rtPA) and then transported to a hospital in a helicopter. The helicopter typically presents an unavoidable vibration in the range of 0.5–120 Hz. Researchers [68,69] have been concerned that the vibrating transport might have a negative effect on outcomes and so studied the effects finding that there is no negative effect. However, they did not consider that there might be a positive outcome and did not record the dominant vibration frequencies or control for them. Most recently Dhanesha et al. [70] did consider the possibility of a positive effect and noted vibration frequency, used a ground-based vibration simulation arm in the study along with the no vibration control and the actual helicopter flight. They found that the animals receiving the rtPA and the simulated vibration had significantly smaller infarctions (areas of tissue death from a blood clot) and increased positive neurological outcomes (86% vs. 28%). Oddly, the actual helicopter flight group did not have significantly positive outcomes which the researchers speculate could be due to the 20–30 Hz band of vibration was attenuated in this helicopter flight. Dhanesha et al. concluded that low frequency vibration is synergistic with rtPA and that application of vibration might be a safe and simple intervention for stroke.

## 3. Neurological Effects

Effects of sound vibration stimulation on the neurological system are many and wide-ranging with multiple complex mechanisms involved. Most of these mechanisms are not yet fully understood but we will point to how the mechanisms at a cellular level seem to function and are related to sound vibration.

### 3.1. Basic Mechanism: Protein Kinases Activation

One of the questions of neuroscience is whether there is a way to regenerate or repair neural damage from neurodegeneration, stroke, transected nerve ends, etc. Electrical stimulation [71] is one intervention that has shown some success in creating axonal regeneration and neurite outgrowth. Low frequency sound vibration also appears to have potential at stimulating neurite outgrowth and neuronal differentiation.

Koike et al. [72], motivated by the intent to find why music therapy might be useful for Alzheimer’s disease (AD) patients, conducted a study to determine if vibratory sounds might enhance neurite outgrowth. They focused on an in-vitro examination of PC12m3 cells known to be sensitive to nerve growth factor (NGF) that induces differentiation of nerve cells and neurite extension. They looked specifically at the p38 mitogen-activated protein kinase (MAPK) activity that has been shown by research with electrical stimulation [71] to be a pathway to enhancing PC12m3 cell growth, and which also appears enhanced in AD. They found that vibratory sound in the 10–100 Hz range had a positive effect on neurite growth with the strongest effect being at 40 Hz whereas vibratory sound at 150 Hz and 200 Hz had little effect. They found that 40 Hz stimulation enhanced p38 MAPK activity indicating that the neural outgrowth they observed was induced through the p38 MAPK pathway.

Cho et al. [73] and Kim et al. [74] examined the effect of 40 Hz sonic vibration on cell differentiation of human umbilical cord-derived mesenchymal stem cells (hUC-MSCs). hUC-MSCs can differentiate into various types including neurons. Cho et al. applied vibration to the cells continuously for 5 days; Kim et al. for 3 or 5 days. Cho determined the differentiated cells were neuron-related oligodendrocytes because they expressed microtubule-associated protein 2 (MAP-2), which is a marker of synapse plasticity, glial fibrillary acidic protein (GFAP) thought to maintain astrocyte structure, and myelin basic protein (MBP) a component of myelin structure. Cho determined that the mechanism mediating the effect of vibration on the UC-MSCs was the extracellular-signal-regulated kinase (ERK) pathway. Kim et al. similarly examined protein expression related to neural differentiation stimulated by the 40 Hz vibration. They found that the protein calponin 3 (CNN3) promoted functional neural differentiation.

Choi et al. [75] examined the effect of multiple frequencies of low frequency vibration on adipose tissue-derived stem cells (AT-MSCs). Adipose tissue is commonly referred to as body fat and AT-MSCs are among the most studied in recent research. Choi applied vibration to the AT-MSCs for four days at 10, 20, 30, and 40 Hz with specific focus on the mechanism of ERK. They found that 30 Hz was most effective at affecting neural differentiation of these specific stem cells.

### 3.2. Basic Mechanism: Nerve Stimulation

Evidenced-based research repeatedly shows positive clinical effects from the application of pulsed stimulation of the body. This applies in varied neurological conditions including cerebral palsy [76], multiple sclerosis [77], and chronic musculoskeletal pain [78]. The question is whether this results in some way from a stimulation of the nervous system and if so, how does that occur. How pervasive is a system to respond to vibration? In the living body transmission of sensory information depends on sensory neurons and mechanosensation at axonal terminals in peripheral nerves. Different types of sensing neurons include mechanosensors detecting external signals, proprioceptors receiving internal body signals, and many types of nociceptors that detect noxious body-threatening stimuli. Usoskina et al. [79] examined the molecular mechanisms activated in the process of cells detecting vibration. By observing calcium ion transients in the somata of neurons, they saw that neurons reliably detected every individual stimulus (e.g., each molecular compression in a sound wave) and then converted these into specific firing patterns in the nerves. Given this basic mechanism, we next look at several categories in which this is applied.

#### 3.2.1. Submechanism: Sensitization of the Proprioception System

The body’s proprioception system gathers and processes information about changes in the position of joints and limbs and, therefore, is strongly involved in the control of posture and movement. Proprioceptors are mechanosensory neurons in the skin (Merkel disks and Meissner’s and Pacinian corpuscles), muscles (spindles), tendons (Golgi tendon organs), and joints. This proprioception system involving receptors, nerves, spinal cord, and pathways of the central nervous system terminates within the thalamus and cerebral cortex [36].

The proprioception system is very sensitive to vibration and, since it is an important factor in motor control, the effect of vibration has been the subject of considerable research, especially the effect of whole body vibration on the rehabilitation of neurological disorders [80]. The stimulation or sensitization of the proprioception system appears to engage a mechanism that retrains the body-mind strategies of motor control or establishes greater consonance between input from the senses and output to the motor system at the cortical level [81]. Perhaps pointing to a cascade effect in this proprioceptive mechanism, which is not fully understood, Delecluse et al. [82] propose that vibration may enhance corticospinal cell connectivity to spinal motor neurons.

##### Application: Complex Regional Pain Syndrome (CRPS)

Gay et al. [81] postulated that CRPS type I may be caused by a sensory input–output mismatch that leads to motor programming disorganization in cortical structures. They hypothesized that enhancing proprioceptive feedback with vibratory stimulation would minimize pain and increase range of motion. The study by Gay et al. [81] applied sinusoidal vibration at 86 Hz to the hand and wrist of patients with CRPS for 20 min a day, five days a week for 10 weeks in addition to conventional rehabilitation sessions. The control group received only the conventional treatment. The results showed that pain severity was lower by close to 50% and range of motion improved by about 30% in the treatment group. They attributed this result to a reestablishment of sensory input–output consonance.

##### Application: Cerebral Palsy

The research of Katusic et al. [80] proceeded on the premise that proprioception is crucial to motor control and hypothesized that sound-based vibration can resonate through the body and enhance sensation of body position, location, and orientation. Further they accepted the premise of Delecluse et al. [82] that vibration could alter corticospinal cell and spinal motor neuron connectivity and that this stimulation of the proprioceptive pathways could rearrange motor control strategies resulting in better postural stability. To test this Katusic et al. [80] did a three month study with 89 children with spastic cerebral palsy (CP) randomized to a physiotherapy only group and to physiotherapy plus vibration group. The vibration treatment, applied with a mat they could lie on, consisted of 40 Hz sine waves for 20 min, two times a week for 12 weeks. The vibration treatment group improved significantly in both spasticity and in gross motor function.

Ko et al. [83] observe that vibration has recently been shown to improve proprioception and thereby balance and motor skills. They postulate that this may be because vibration stimulates muscles and tendons. To test whether whole body vibration at 20–24 Hz would affect sense of joint position, gait, and balance in children with CP, they randomized 24 children to physical therapy (PT) or traditional PT plus vibration for 20 min (3 min on, 3 min off) two times a week for three weeks. They found significant improvement in joint position sense and improvement in gait variables in the vibration group.

#### 3.2.2. Submechanism: Vagal Nerve Stimulation

The vagus nerve, one of the 12 cranial nerves, serves as a major parasympathetic (efferent) component of the autonomic nervous system and importantly transmits sensory information from much of the body to the brain [84]. It plays a key role in cardiac and gastrointestinal function, in muscle control of mouth and throat, in the neuroendocrine-immune system, and in the regulation of emotion including anxiety and depression. Vagus nerve stimulation (VNS) [84] is a recognized practice commonly done with manual massage or compression, electrical stimulation, or vibration including with the voice or gargling throat or with external vibrotactile devices. However, the spleen has nerve fibers that are integrated with the vagus nerve and studies [85,86] show that anti-inflammatory effects of the vagus nerve rely somewhat on the splenic nerve to the extent that stimulation of the splenic nerve results in immunosuppressive effects comparable to VNS [87]. Vibration at the abdominal level [88] may then be stimulating the splenic–vagal nerve system. Specific applications of VNS include refractory epilepsy, depression, and decreasing inflammation. One of the known mechanisms by which stimulation of the vagus nerve has its effect is the release of the neurotransmitter acetylcholine.

##### Application: Depression

Sigurdardóttir et al. [88] conducted a study with 38 people with depressive disorder (18 treatment, 20 control) using relaxing music with a specifically created low frequency sound track that activated a vibrotactile transducer at the abdominal level at the back of the chair in which they were seated. The premised mechanism for their intended effect was the activation of Pacinian corpuscles sending an afferent impulse in the vagus nerve to the regions of the brain associated with depression. The vibratory stimulation treatment was applied for 20 min in eight sessions over 3–4 weeks. The authors did not report what specific frequencies they employed but maintain that Pacinian corpuscles stimulated at 240 Hz have a maximal afferent output but afferent output occurs at any frequency below that. Although not a rigorously controlled study and not measuring changes in the vagal tone, the pilot study did find a reduction in depression scores in the treatment group and attributes this to stimulation of the vagus nerve and the central nervous system through the abdomen. A study by Braun Janzen et al. [89] that applied a very similar treatment also found a reduction in depression and anhedonia although it did not premise vagal stimulation.

##### Application: Rett’s Syndrome

Rett’s syndrome (RS) is a gene mutation-based neurological disorder mainly affecting females that involves brain stem immaturity [90] resulting in dysfunction of the autonomic nervous system. The resultant symptoms are numerous with many associations to the vagus nerve. These include breathing and cardiac function, speech and hearing-related communication, and movement control and seizures [91,92]. Vagus nerve stimulation has shown positive results in reducing epileptic seizures [91] and along with auditory stimulation has been effective in increasing cortical strength and auditory function [92].

Bergström-Isacsson et al. [93] examined neurophysiological brainstem data and emotional response in 29 Rett’s participants and 11 controls to six stimuli with music and low frequency vibration at 40 Hz. Each stimulus was presented for 10 min in a repeated measures design. Outcome measures used were mean arterial blood pressure, the coefficient of variation of mean arterial blood pressure, cardiac sensitivity to baroreflex, and cardiac vagal tone (CVT). Results showed that the strongest parasympathetic response in the Rett’s group was to the 40 Hz vibratory stimulation and with a significant increase in CVT.

### 3.3. Category of Neurological Effects—Pain and Vibratory Analgesia Specific

The pain-reducing effect of vibration is demonstrated in numerous evidence-based research studies [78,94,95,96,97]. Mechanical and acoustic vibrations have been used extensively to address pain and is a treatment technique in orthopedics and low back pain [98,99], physiotherapy [100,101,102], during cosmetic procedures [103], and during orthodontic work and orofacial pain [104,105,106,107]. However, until relatively recently, very little was thoroughly described and understood about pain perception mechanisms. Studies with electrophysiology have shown cortical neurons responding to noxious stimuli, but it has not become clear to what extent this response represents pain or correlates with it [108]. Consequently, the mechanisms by which vibration acts as an analgesic is less understood.

#### 3.3.1. Basic Mechanism: Gate Control

One of the submechanisms of the general mechanism of nerve stimulation but a basic mechanism of analgesia is focused on the function of the substantia gelatinosa in the dorsal horn and is commonly known as gate control. The theory for this pain mechanism was proposed by Melzack and Wall [109] and postulates that the substantia gelatinosa modulates sensory information being transmitted to the spinal cord and the brain. Specifically, signals from pain receptors are carried to the dorsal horn by small diameter afferent A-delta and C fibers. The signal transmission from the pain receptors can be modulated (inhibited) by the large afferent A-alpha and A-beta fibers transmitting sensory signals from skin sensation such as touch or vibration [78,96,109]. This mechanism known as gate control theory has been subject of considerable criticism and aspects have been questioned [110,111] and enhanced by Melzack’s neuromatrix of pain theory [112] but its fundamental function remains.

Salter and Henry [113] specifically explored the response of wide dynamic range (WDR) spinal neurons in the lumbar dorsal horn to vibration at different amplitudes and frequencies to determine how they might play a role in analgesic effects. They tried a variety of frequencies and intensities and found that WDR neurons entrained to the vibratory frequencies below 80 Hz. Their findings suggest that pain reduction is accomplished by the effect of vibration on Pacinian corpuscle afferents and the WDR neuron response in the dorsal horn but that frequency, location, and intensity of the vibratory stimulus is a factor that needs further clarification.

#### 3.3.2. Basic Mechanism: Modulating Autonomic Responses—Pain and Beyond

An alternative medical practice that has growing scientific evidence for its effect on the central nervous system and regulating autonomic responses is acupuncture [114]. The clinical efficacy of acupuncture may reside in its neurobiological foundations and in its purported effect of creating biochemical changes in the body and brain that control the autonomic nervous system’s functions such as blood pressure, heart rate variability, skin temperature, and perception of pain. The detailed explanation of mechanisms is still needed.

One question that arises in relation to acupuncture is whether the specific acupoints must be punctured with needles or whether vibratory sound stimulation might have the same effect. Xu et al. [115] explored this question by looking for microcirculation using laser Doppler flowmetry at meridian acupoints while stimulating the points with low frequency sound waves between 16 and 160 Hz. They found different points respond optimally to different sound frequencies. For example, the point Yin Ling Quan: A0 responded to 29.14 Hz, Zusanli A1 responded best to 58.27 Hz, and Tianjing A2 to 110.00 Hz. Brătilă and Moldovan [116] used an electrical input output model to determine optimal frequencies for the lung meridian (124 Hz) and kidney meridian (120 Hz). Weber et al. [117] explored the use of a simultaneous sound combination (32, 48, and 64 Hz) for its effectiveness to reduce perceived pain intensity and pain threshold compared to a single frequency sound stimulation and a sham. They applied electromagnetic transducers to five acupoints indicated for relief of pain and anxiety and subjected 13 pain-free participants to a cold pressor test. Results showed that both multifrequency and single frequency sound stimulation significantly improved pain tolerance and reduce intensity of pain.

#### 3.3.3. Basic Mechanism: Neurotransmitters—Pain and Beyond

Unpacking the complexity of the role of neurotransmitters and neuromodulators on the body and brain is beyond the scope of this paper. However, research has shown instances in which neurotransmitters are stimulated by vibration and these will be described here.

In relation to vibratory analgesia, Salter and Henry [118] examined specifically the vibratory activation of P_1_-purinergic receptors in the dorsal horn by the neurotransmitter adenosine. In a study with cats, vibration was applied at 80 Hz in 2.5–3.5 s trains every 20–25 s for 10 min. This resulted in a vibration-induced depression of lower lumbar nociceptive neurons and remained in effect for up to 4 h after the stimulation. Various agents used to attenuate the depression of these neurons revealed that adenosine was responsible for the analgesic effect. The study suggests that the effect earlier described as gate control may be mediated by the release of adenosine resulting from vibration.

Several neurotransmitters not specifically related to pain have already been discussed in other contexts above. Assuming vibration can stimulate the vagus nerve and the related splenic nerve as discussed above, vibration then has the effect of releasing acetylcholine, which plays a key role in synapses and especially at the location where nerves and muscles connect. It also plays a role in controlling the autonomic nervous system and a particularly important role in the cognitive system and in regulating heart rate. Additionally, stimulation of the splenic nerve causes the induction of norepinephrine. Nitric oxide, extensively discussed above, is also a neurotransmitter serving to regulate and mediate processes of the nervous, immune, and cognitive systems.

Gamma-aminobutyric acid (GABA) is a prominent neurotransmitter in the brain and the central nervous system. It plays a crucial role reducing the activity of neurons and especially in control of fear and anxiety. Safarov and Kerimov [119,120] in two separate animal studies explored the effect of low frequency vibration (20 Hz) on GABA levels and its metabolism. The found that vibration, regardless of duration, increased GABA level in the brain stem, the large hemispheres, and the cerebellum as well as activity of the glutamate decarboxylase enzyme, which produces GABA. However, they found the effect more marked with 30 min stimulation than with long periods like 7 h. The implication is that one of the mechanisms creating the relaxation effect of vibroacoustic stimulation may be the boost in neurotransmitter GABA.

### 3.4. Basic Mechanism: Oscillatory Coherence Supports Connectivity and Circuit Function

The neurological effect from rhythmically pulsed sensory stimulation is premised on two important postulates: (1) that rhythmic sensory stimulation (RSS) drives a neural response resulting in increased oscillatory coherence and (2) that oscillatory coherence is crucially linked to connectivity, circuit function, and related to health conditions.

As to the postulation that RSS drives a neural response, recent research in somatosensory, auditory, and visual modalities shows that vibrotactile rhythmically pulsed stimulation has a strong neural driving effect [121,122,123]. For example, vibratory stimulation of a finger, the hand, or the median nerve results in an oscillatory response in primary and secondary sensorimotor cortices [124,125,126] and attention plays almost no role [127]. More auditory rhythmic stimulation research has been done to elicit steady-state or spontaneous oscillatory responses using clicks, amplitude-modulated isochronous sounds [128], or pure tones. Examples include a 40 Hz amplitude modulated tone [129,130], or even the rhythms of binaural beats that are created through binaurally detuned tones [131]. What follows then is the conclusion that RSS can drive oscillatory coherence.

The assertion that oscillatory coherence is linked to circuit function and related to health conditions is more crucial but less understood. Deep brain stimulation (DBS) research suggests that circuit dysfunction is common to many psychiatric and neurological conditions [132,133]. Basically, the dysregulation of circuits that underlies these conditions stems from a lack of excitation-based coherence, disturbed coherence, or coherence that is overly strong in inappropriate neural populations. Llinas pointed specifically to the recurrent connections between the thalamus and the cortex that have a mechanism function to connect areas of the cortex and control information flow [133,134,135,136,137]. These thalamocortical loops serve communication in the brain similar to what an internet hub does. Llinas further maintained that thalamocortical interconnectivity depends primarily on rhythmic oscillatory activity. Thalamocortical loops function optimally with rhythmic oscillatory activity in the cortex in the gamma band (40 Hz) and in the thalamus in the alpha band (10 Hz). Thalamocortical dysrhythmia (TCD) is characterized by a decrease in alpha band activity (power) with a related increase in theta band activity (4–7 Hz) and a reduction in cortically consistent gamma band activity. TCD appears related to neurological and psychiatric conditions; specifically cognitive, motor, auditory, and mood functions. TCD is linked to conditions including Parkinson’s disease, major depression, neurogenic central pain, tinnitus, and schizophrenia [134].

Assuming then that vibratory stimulation (RSS) can drive oscillatory coherence and potentially regulate dysrhythmic circuits in the brain, RSS may employ the mechanism of oscillatory coherence and positively affect the health conditions resulting to some extent from these dysrhythmias. The positive response of major depression to RSS may be an indicator of this [89]. The mechanism of driving oscillatory coherence with RSS broadens the focus from the neuroscience of circuit connections (the connectome) to the framework of dynamic brain rhythms related to neural spiking activity (the dynome) [13].

#### 3.4.1. Application: Neurogenic Pain

Some mechanism for vibratory analgesia were explained above under gate control. However, there is pain that does not appear to stem from nociception and where the application of vibratory stimulation cannot be affecting only the dorsal horn neurons because the frequency is above the primary response level of Pacinian corpuscles and the rapidly adapting mechanoreception system. Such pain may then be neurogenic, stemming from neural circuit dysrhythmias or disconnections. Hollins et al. [97] explores this and determines that in some cases the vibratory analgesia must stem from cortical dynamics and specifically from an interaction of Brodmann areas 3a and 3b/1. Fallon et al. [138] observed that in Fibromyalgia (FM) patients there was increased theta power in the prefrontal and anterior cingulate cortices consistent with TCD characteristics. This increased frontal theta activity was significantly correlated measured tenderness and tiredness scores. Jensen et al. [139] used brain imaging to examine brain connectivity in FM patients and found less connectivity between the rostral anterior cingulate cortex (rACC) (known to play a role in inhibiting pain) and the hippocampus extending into the part of the brain stem known to modulate pain.

In this context where FM seems to be related to brain dysregulation and connectivity, where pain appears to be related to brain region interaction, and where there is demonstrated potential for vibration to drive neural coherence, it can be speculated that the positive results from vibratory stimulation on FM patients were due to the mechanism of oscillatory coherence [30,140]. Both studies [30,140] used 40 Hz vibrotactile stimulation of the body, used oscillatory coherence and its relation to connectivity as a premise for effect, but did not establish mechanism with brain imaging. The authors are currently engaged in a study to explore this mechanism.

#### 3.4.2. Application: Neurodegenerative Conditions

The specific mechanisms or causes related to neurodegenerative diseases and conditions are not fully understood but one path being explored with some success is that of spontaneous brain oscillation power, synchronization, dysrhythmia, and circuit connectivity.

Dockstader et al. [141] studied the oscillatory power in various frequency bands in brain regions using magnetoencephalography (MEG) imaging in children who had been treated for brain tumors with cranial radiation therapy (CRT). Children treated with CRT consistently are found to have cognitive deficits in attention, processing speed, and memory. The results showed a clear pattern of gamma deficits with a significantly lower gamma oscillation power in the 60–100 Hz range in the resting state.

Stam et al. [142] studied Alzheimer’s patients (AD) and healthy controls with full-head MEG with the purpose of investigating linear and non-linear interdependencies in the MEG signal channels. Results showed that this measure of synchronization was lower in AD in the 10–14 Hz alpha band, 18–22 Hz beta band, and in the 22–40 Hz gamma band. Additionally, studying AD patients with MEG, Ribary et al. [136] looked at 40 Hz oscillatory coherence at cortical and subcortical sites associated with the thalamocortical loop. Results showed that although AD patients had similar activity patterns as healthy patients, the AD patients had reduced gamma activity in the cortical component.

Recent research [42,43,143,144,145] demonstrated that 40Hz auditory, vibrotactile, or visual rhythmic sensory stimulation (RSS) had significantly positive results on AD symptoms. Although none of these studies confirmed the effect on oscillatory coherence in humans, that mechanism can be inferred from the method and results. A possible submechanism of RSS driving oscillatory coherence at 40 Hz was shown by Iaccarino [42]. They demonstrated that in AD mice the effect of the general 40 Hz RSS was to drive fast-spiking parvalbumin-positive-interneurons (at 40 Hz but not at other frequencies) and as a result reducing amyloid-β levels. They also observed a reduction in inflammation, increase in microglia activity, blood vessel lumen size, and cognitive performance.

Lozano and Lipsman [132] review and explain dysfunctional brain circuits and specifically how deep brain stimulation (DBS) can mitigate the effect, for example, of the motor circuit affecting Parkinson’s disease (PD). The role of DBS in relation to the dysfunctional motor circuit in PD is the inhibition of a group of neurons with 130 Hz electrical stimulation. While the widely accepted approach of DBS is an inhibitory one, Neuman et al. [146] point out that DBS modulates the motor circuit but does not restore it functionally. They differentiate the motor circuit pathways more precisely in the basal ganglia and suggest their work may inspire innovative ways to improve the therapeutic efficacy of neuromodulation in PD. Several studies have used sound vibration as a neuromodulatory stimulant with PD patients [44,147]. One study applied 30 Hz and the other 40 Hz. Both had significant positive results and, although no neuroimaging was used to verify effects on circuit function, they pointed to the potential mechanism of vibration therapy with PD.

#### 3.4.3. Submechanism: Frontal Oscillatory Symmetry

The discussion above has already identified a vibration mechanism related to major depressive disorder (MDD) under vagal stimulation and under oscillatory coherence and TCD. Decreased connectivity is a clearly identified factor in MDD [148] and so affecting MDD by driving coherence with rhythmic sound and vibration is a possibility. There is a further potential mechanism for MDD related to frontal asymmetry. There is considerable evidence that frontal EEG asymmetry between left and right in the alpha band is a biomarker for depression and anxiety [149] but age, gender, and severity of depression interaction raises a caution on this [150]. However, studies have shown that using music stimulation with depressed participants has resulted in a reduction of frontal asymmetry in the EEG [151,152,153]. These music studies did not use low frequency sound or vibrotactile stimulation. The only known study that has used vibrotactile gamma stimulation [89] did show significant reduction in depression severity. More research into the occurrence of frontal oscillatory asymmetry and vibratory stimulation is needed.

## 4. Musculoskeletal Effects

Effects of sound vibration stimulation on the musculoskeletal system are many and wide-ranging with multiple complex mechanisms involved. These structures include the muscles, skeleton, intervertebral discs, ligaments, and other associated structures. We will point to how the mechanisms at a cellular level seem to function and are related to sound vibration and how it translates to applications in humans.

### 4.1. Basic Mechanism: The Muscle Stretch Reflex

The physiological basis for vibration acting on the muscles involves the mechanical stimulation of the muscle stretch reflex leading to neuromuscular potentiation [154]. The reflex functions to maintain a constant muscle length so any stretches result in involuntary muscle contractions, and so a low frequency (0–200 Hz) paradigm of vibration can lead to thousands of such muscle contractions within minutes of application [155]. Vibration of the muscles causes a cascade of events: afferent neurons stimulate alpha motor neurons, leading to motor unit recruitment, increased firing frequency, and/or improved synchronization, which leads to quicker or more forceful muscle contraction and an overall increase in muscle fibers over time (i.e., hypertrophy) [154]. Muscle hypertrophy is correlated with an increase in protein synthesis and an addition of contractile filaments, leading to greater muscle strength. The Akt/mTOR/p70S6K signaling pathway in muscle cells is a crucial component in the hypertrophy process and is necessary in inhibiting the opposite (i.e., muscle atrophy), and has been shown to be enhanced by vibration stimulation in both mechanical loading and muscle injury contexts [156]. Several studies have shown that Akt levels increase in response to muscle contractile activity and mechanical tension, both of which are stimulated by vibration treatment [155,157].

Vibratory stimulation of muscle cells in vitro and in vivo also decreases the expression of genes that prevent atrophy: namely myostatin and atrogin-1 [158,159]. Myostatin regulates muscle growth by inhibiting myogenesis and atrogin-1 enhances proteolysis functions. The decrease in myostatin and atrogin-1 after 30 Hz vibration treatment has been associated with the fusion of satellite (muscle) cells [155].

The muscle hypertrophy pathway and atrophy pathway are not completely independent however, and function antagonistically to each other. For example, when Akt is activated it phosphorylates FOXO1 and sequesters it in the cytoplasm, inhibiting its transcriptional activity [156]. Without Akt activity, FOXO1 can induce transcription of atrogin-1, which leads to atrophy via increased protein degradation [160,161]. Vibration may also enhance mitochondrial biogenesis, which normally occurs as a major adaptation of skeletal muscles in response to exercise training. One study found that vibration at 50 Hz enhanced the expression of PGC-1α in the soleus muscle, gastrocnemius muscle and liver, and was associated with increased muscle strength [162]. PGC1α plays an important role in mitochondrial biogenesis and is regulated by mitogen-activated protein kinase p38 (p38 MAPK) and is activated during exercise.

#### Application: Using Vibration to Provide Exercise Like Effects

The use of vibration as a tool to produce exercise-like effects on the muscles provides valuables applications in the rehabilitation sciences. The most well-known use of vibration is for promoting muscle recovery and performance in athletes [154,163,164]. However, there are lesser known uses that are crucial for the treatment of a variety of medical conditions, especially in situations where exercise is necessary but difficult to achieve. We shall present three such examples to follow.

Gloeckl et al. [161] and Lage et al. [162] were able to safely and successfully use vibration therapy as a pulmonary rehabilitation tool for patients with chronic obstructive pulmonary disease (COPD) who require physical exercise but cannot perform due to breathing limitations and general malaise. Although physical exercise is the basis of pulmonary rehabilitation for people with COPD, intense exercise may also stimulates pro-inflammatory cytokines and can be harmful in that context [165,166]. Since the magnitude of the exercise stimulus is related to the increase in inflammatory cytokines, treatment for COPD needs to stimulate the muscles without such complications [167]. For this reason, clinical studies have tested vibration therapy for COPD and found functional improvements in mobility and an anti-inflammatory effect [168,169].

Another application has been in the use of vibration treatment for frail elderly people. Sarcopenia is a form of muscle loss that occurs with aging and/or immobility. Strength training can reverses age-related muscle and strength losses while promoting muscle hypertrophy [170]. However, due to the intensity of exercise needed to achieve such a result the appeal and application by the elderly is very restricted, with only 10–15% of this population reported to engage in such training [171]. Vibration treatment of the elderly is thus a safe and effective strength training tool that has shown high compliance among the elderly population and has shown positive results in improving in balance, physical function, and muscle strength [172,173,174].

A third condition for which vibration may be applied is for Duchenne muscular dystrophy (DMD), which is a degenerative disorder caused by a defective gene responsible for producing a muscular protein called dystrophin. This protein plays an important role in preventing muscle fatigue, and patients with DMD often have poorly developed muscles and, as a consequence, bone. Exercise is a crucial treatment for muscle and bone growth; however, over-activity among DMD patients can result in pain, myoglobinuria, and further fatigue in the muscles [175]. Therefore, vibration treatment can act as a useful adjunct to exercise therapy to keep muscle fatigue low while maintaining optimal growth. Moreira-Marconi et al. (2017) found that vibration treatment can improve or maintain functional mobility and strength in the muscles among DMD patients [176]. In summary, the benefits of vibration on the muscles present an important modality for treatment that includes a low risk, high compliance form of stimulation that may benefit a wide variety of conditions affecting the musculature.

### 4.2. Basic Mechanism: Determining Bone Cell Progenitor Fate

The skeleton is a very dynamic structure that is maintained by bone deposition and resorption, determined largely by the activity of osteoblasts and osteoclasts respectively. Structurally, bone is created of different types of cells and a rich extracellular matrix (ECM). Osteoblasts have the ability to secrete and calcify the ECM and are derived from mesenchymal stem cells (MSCs). The osteogenic differentiation of MSC’s involve three stages: commitment to osteogenic lineage, matrix synthesis, and matrix mineralization [177]. The major genetic markers for osteoblasts include RUNX2, ALP, Col-I, OSX, and OCN.

(1) RUNX2: a protein encoded by the RUNX2 gene that is a key transcription factor associated with osteoblast differentiation.
(2) ALP: alkaline phosphatase. An enzyme responsible for breaking down proteins and is associated with bone growth.
(3) Col−1: collagen type 1. It is main structural protein in the extracellular matrix found in the body’s various connective tissues.
(4) OSX: Osterix, also called transcription factor Sp7. It plays a major role in driving the differentiation of mesenchymal precursor cells into osteoblasts and eventually osteocytes.

Zhou et al. (2011) investigated the effect of vibration on the osteogenic differentiation of MSCs seeded on human bone-derived scaffolds and found that 40 Hz vibration promoted MSC differentiation by upregulating the mRNA and protein expression of RUNX2, ALP, Col-I, and OCN [178]. Zhang et al. (2012) cultured periodontal ligament stem cells under vibration and in the 40–120 Hz range found increased levels of ALP, Col−1, Runx2, Osx, and OCN [179]. Additionally, Prè et al. treated MSCs with mechanical vibration, and the results showed that the expression of ALP and Runx2 was significantly increased after 30 Hz mechanical vibration treatment (2017) [180]. This suggests a direct role in vibration in influencing the stem cell fate toward osteoblast formation.

RUNX2 and OSX are usually highly expressed at the early stages where stem cells commit to the osteogenic lineage [181,182], Col−1 and ALP at the middle stage (matrix synthesis) [179,182], and OCN at the late stage of osteogenesis [183]. RUNX2 and OSX are transcription factors that are important for stem cell commitment to the osteogenic lineage and are expressed specifically in osteoblasts. RUNX2 are active earlier and OSX at a later step once preosteoblasts form into fully functional osteoblasts. Studies looking at molecular and genetic manipulation of RUNX2 in vivo found that its expression is necessary for MSC differentiation towards the osteoblast lineage [184,185], and without it this process cannot begin [186]. RUNX2 is also known to negatively regulate osteoblast proliferation by acting on the cell-cycle [181], and prevents terminal differentiation of osteoblasts to keep them in an immature state. This corresponded with several studies that found that the proliferation of osteoblasts did not change or was decreased after vibration [187,188,189]. OSX is a more specific marker of osteoblasts and is expressed after RUNX2, and further guides differentiation from preosteoblast to immature osteoblast state [182]. OSX binds to the nuclear factor of activated T cells (NFAT), which leads to the expression of collagen type 1 and activation of the Wnt signaling pathway [190]. Chen et al. (2016) found that vibration treatment of MSCs cultured on hydroxyapatite-coated surfaces increased in the expression of Wnt and β-catenin in addition to RUNX2 and OSX [177]. The Wnt/β-catenin signaling pathway is a normal physiological response to mechanical loading, which enhances the sensitivity of osteoblasts to further mechanical loading [191]. Hou et al. (2011) investigated the mechanisms of vibration-enhanced osteogenic responses in MC3T3-E1 cells and they demonstrated that Wnt signaling was involved in the transduction of vibrations, which caused a decrease in the RANKL/OPG ratio and levels of sclerostin [192]. The RANKL/RANK/OPG pathway is an important signaling pathway in the formation and activity of osteoclasts, and is one that is also influenced by vibration treatment. Osteoclasts are derived from monocytes and macrophages, which proliferate in the bone marrow and fuse to give rise to multinucleated osteoclasts [193]. Osteoclast progenitors express RANK (receptor activator of nuclear factor-κB), which interact with the RANK-ligand (RANKL) to initiate osteoclastogenesis. RANKL is produced by a variety of cells such as osteoblasts, osteocytes, stromal cells, and lymphocytes. Osteoprotegerin (OPG), on the other hand, binds competitively to RANK and inhibits this process. Therefore, the availability and interaction of RANKL/RANK/OPG determine the rate of osteoclastogenesis [194]. Therefore, the Wnt/β-catenin signaling pathway stimulated by vibration produces a dual function to stimulate the formation of osteoblasts while inhibiting the formation of osteoclasts. In other words, vibration stimulated an increase in bone amount and quality.

Kulkarni et al. (2013) found that mechanical vibration of osteoclast precursor cells reduce DC-STAMP expression in the presence of RANKL in osteoclast precursor cells leading to the inhibition of osteoclast formation [195]. Wu et al. (2012) found that stimulation of murine monocytes by vibration reduced the expression of osteoclast-specific genes such as cathepsin K, matrix metallopeptidase-9 (MMP-9), and c-Fos to such a level that cells could not differentiate into osteoclasts even in the presence of RANKL [196]. Kim et al. (2012) and Sun et al. (2011) independently found that vibration stimulation of mesenchymal stromal stem cells increased OPG expression, indicating a direct effect of vibration in inhibiting osteoclast formation [197,198].

In summary, it seems that vibration stimulation on bone related stem cells promote anabolic processes by stimulating the formation of osteoblasts while simultaneously inhibiting catabolic processes by inhibiting the formation of osteoclasts.

#### Application: Tissue Engineering

A major application of vibration in the differentiation of stem cells in the context of bone health is in engineering bone tissue. Typically, this includes osteoblasts and other bone cell progenitors growing and attaching to an artificial surface for eventual implantation. The ability of MSCs to adhere to the implant surface and their differentiation on the implant surface are important components of successful osseointegration [199,200]. In order to analyze cell adhesion to the implant surface, Chen et al. (2016) assessed the matrix organization and the cytoskeleton rearrangement (F-actin), and the gene expression of F-actin, β1 integrin, vinculin, and paxillin, which are involved in the adhesion of cells to substrates. They found that vibration of MSCs on hydroxyapatite-coated surfaces significantly increased the expression of F-actin, and such upregulation of F-actin by mechanical stress has also been reported in other studies [201,202]. Mechanical strain is known to induce changes in cytoskeletal organization, and actin filaments are crucial for cell adhesion [203]. Chen et al. (2016) also found an increase in other proteins necessary for adhesion such as β1 integrin, vinculin, and paxillin [177]. This has been found in other studies as well [201,204]. In summary, the differentiation of MSCs to anabolic processes and the adhesion to surfaces after vibration allow for this to be an ideal application for engineering bone tissue in the lab for implantation.

### 4.3. Basic Mechanism: Vibration Effects on Ossification and Resorption

Bone remodeling is a lifelong process where mature bone tissue is removed (i.e., bone resorption) and replaced by new bone tissue (i.e., ossification). This process is important for the adaptation of bone to mechanical loading and healing from microdamage and fractures. The imbalance of bone resorption and ossification leads to disorders such as osteoporosis, a systemic skeletal disorder characterized by the deterioration of bone tissue leading to bone fragility and an increase in fracture risk. Bone mineral density (BMD) is a measurement of the amount of bone mineral in bone tissue, and is used clinically to assess the risk to osteoporosis or fracture. In this section we lay out the evidence that vibration treatment has anabolic effects on bone remodeling, and how this translates to better bone health and BMD when applied to humans.

Healthy bone remodeling involves maintaining a balance between bone resorption and ossification and is maintained by a network of regulatory proteins [205]. Bone resorption is done via the action of osteoclasts that break down bone, and bone formation (i.e., ossification) is mediated by osteoblasts, which secrete new bone material. As described in Section 4.2, vibration stimulation has been showed to shift the balance towards bone formation by enhancing anabolic pathways and inhibiting catabolic pathways. Osteocytes (i.e., mature bone cells) are the major mechanosensor in bone that influence osteoblast and osteoclast activities when subjected to a variety of mechanical stimuli, including fluid flow, hydrostatic pressure, and mechanical stretching [206].

Animal models have shown that vibration at 10–100 Hz can stimulate bone growth by doubling bone formation rates and inhibiting osteoporosis [207]. Bacabac et al. (2006) specifically tested the effects of vibration on osteoblast precursor cells at a variety of frequencies from 5 to 100 Hz. They found that nitric oxide (NO) release and COX-2 mRNA expression were positively correlated with frequency whereas prostaglandin E_2_ release negatively correlated with frequency [208]. It has been shown in vitro that osteoclasts migrate away from NO [208,209], and PGE2 I stimulates osteoclasts differentiation and activity by increasing mRNA levels of osteoclast differentiation factors. Therefore, the anti-correlation of NO and PGE_2_ has a net effect of stimulating ossification over bone resorption.

Da Jing et al. (2016) tested vibration at 45 Hz and 0.5 g in leptin deficient mice, which exhibit decreased bone mass and impaired bone microstructure. They found that vibration treatment in these rats stimulate tibial alkalinephosphatase (ALP), OCN, runt-related transcription factor 2 (RUNX2), type I collagen (COL1), BMP2, Wnt3a, Lrp6, and b-catenin mRNA expression, and prevented the increases of tibial sclerostin (SOST), RANK, RANKL, and RANL/osteoprotegerin (OPG) gene levels indb/dbmice [210]. These proteins are pro-ossification and antibone resorption, and this ultimately resulted in increased mineral apposition rate, bone formation rate, and increased osteoblast numbers in cancellous bone. These beneficial effects on bone cells are supported by Li et al. (2015), who tested ovariectomized rats with vibration (35 Hz at 0.25 g) [211]. They found that vibration upregulated the expression of BMP-2 and Runx2, activated the ERK1/2 signaling pathway, and consequently led to increased expression of OCN. These proteins are also pro-ossification. They also noted that the anabolic effect of mechanical stimulation was enhanced with the incorporation of resting period between loadings. These findings were replicated by Sun et al. (2019) who showed that MLO-Y4 osteocyte like cells stimulated by 45 Hz at 0.5 g promoted the secretion of prostaglandin E_2_ and OPG, and inhibited the secretion of tumor necrosis factor-α and RANKL [212]. Tanaka et al. (2003) found similar results testing osteoblasts in vitro with vibration [213]. Lau et al. [206] found that stimulation of MLO-Y4 osteocyte cells stimulated with low amplitude high frequency vibration (0.3 g; 30, 60, and 90 Hz) had a variety of antibone resorption effects, which varied with frequency. They found that osteocytes increased the expression of COX-2 at 90 Hz, while RANKL decreased most significantly at 60 Hz. Conditioned medium collected from the vibrated osteocytes cells inhibited the formation of large osteoclasts and the amount of osteoclastic resorption by 20% [206]. Similar findings have been found in other studies and have also found an increase on chondrogenic factors such as collagen type I and II, and have shown corresponding improvements of fractures from radiographs and histomorphometry [214,215]. Computed tomography imaging evidence on fracture healing showed that ovariectomized mice undergoing 45 Hz vibration treatment demonstrated improved callus properties, with increased flexural rigidity and bone formation in addition to the cellular effects as mentioned above [216].

The mechanism behind the frequency dependency of osteocyte response to vibration is not well understood. At lower frequencies (<10 Hz), fluid flow may be what mediates vibration mediated effects, causing calcium oscillations in cells when vibration is applied [217]. Due to the viscoelasticity of cells, cells are less stiff and thus more deformable at lower frequencies. Such deformation caused by fluid shear stress requires contact stress acting primarily on the cell membrane [208]. Consequently, cilia, which are small hair like structures protruding from the cell membrane, are considered important structures for mechanosensation. Li et al. (2020) found that chemicals which damage primary cilia inhibited vibration-induced osteogenic responses (differentiation, mineralization, and maturation) in osteocytes, indicating that vibration-induced osteogenic effects are mediated through cilia [218]. At higher frequencies there is a chance that fluid stress is attenuated, and it is suggested that at frequencies above 10 Hz, the movement of solid intracellular bodies such as the nucleus may drive the cellular effects of vibration. Bacabac et al. (2006) found that the rate of acceleration generated by the vibration frequency was correlated with the amount of force acting on the nucleus of vibrated bone cells.

The effects of vibration may have differing effects on different bones of the body and depending on how vibration is applied. Pravitharangul et al. (2018) found that vibration of bone cells from the iliac had reduced resorptive protein expression but that effect was not found in mandible bone cells [219]. Interestingly, Rubin et al. (2004) showed that whole body vibration signal was maximally transmitted to the hip and spine of people in a standing position [220]. Komrakova et al. (2013) investigated various vibration parameters on fracture healing in mice and found that vertical vibrations of 35 and 50 Hz produced more favorable results than the higher frequencies and horizontal vibrations showed no positive or negative effects. Specifically, vertical vibrations (>35 Hz) improved cortical and callus densities, enlarged callus area and width, accelerated osteotomy bridging (35 and 50 Hz), and increased relative muscle weight (50 Hz) [221].

Estrogen levels may also influence whether vibration generates positive or null effects on bone cells. Haffner-Luntzer et al. [222] found that vibration applied at 45 Hz stimulation of osteoblast cells had either positive bone forming effects of gene expression or a null effect depending on the presence of estrogen. They found that bone cells increased COX-2 gene expression, cell metabolic activity, and cell proliferation after vibration in the absence of estrogen, whereas the contrary was true in the presence of estrogen [222].

This indicates that considerably more research is needed to determine the optimal frequency, amplitude, and direction of vibration for desired bone effects. As well, specific important contextual considerations need to be explored to derive the intended effects of vibrations.

#### Application: Treating Osteoporosis and Bone Loss

An important application of vibration in humans is to stimulate bone healing and growth, especially in response to fractures or osteoporosis. Rubin et al. (2004) performed a prospective, randomized, double-blinded, and placebo-controlled trial of 70 postmenopausal women with brief periods of vibration applied at 30 Hz and 0.2 g [220]. DXA scanning was used to measure bone mineral density in the spine, hip, and distal radius at baseline, 3, 6, and 12 months. They found that vibration treatment significantly reduced bone loss in the spine and femur, with efficacy increasing with greater compliance and for those with lower body mass. Although bone loss was reduced, there did not seem to be any indications of a net increase in bone mass being created. In a more recent study, ElDeeb and Abdel-Aziem (2020) showed that vibration in conjunction with exercise can improve the bone mineral density (BMD) of post-menopausal women with osteoporosis [223]. Other clinical studies have shown similar results to suggest positive effects on osteoporotic BMD [224,225].

Matute-Llorente et al. (2015) performed a randomized controlled trial of adolescents with Down syndrome using whole body vibration, with primary outcomes being bone mineral content and BMD [226]. Adolescents with Down syndrome (DS) tend to have poorer bone health than peers without DS. They found that the vibration treated group increased in bone mineral content and density as a whole and specifically in the lumbar spine area and the tibia. Another randomized, placebo controlled trial found that vibration treatment for children with cerebral palsy improved BMD in tibial regions and the spine compared to controls, even though compliance to using the device was only 44% [227].

Therefore, it can be seen that the beneficial osteogenic effects of vibration on bone health are prominent across different age groups and medical conditions with differing etiologies. What is common is the fact that osteoporosis, down syndrome, and cerebral palsy are disabling conditions that reduce mechanical stimulation of the bones due to a lack of activity or strength in muscles and bone. Since the nature of the vibratory stimulus is based on providing a surrogate for high frequency muscle based signals, this may be used successfully to treat conditions that limit such stimulation naturally. Given that the vibration parameters are constrained to 1–100 Hz at less than 1 g magnitude [228], there is a net beneficial effect of vibration on the bone strength and health in humans.

### 4.4. Basic Mechanism: Anabolic Effects on the Spine and Intervertebral Discs

The spine is an important component of the skeletal system that is crucial for structural integrity of the body and housing the spinal cord, which connects the peripheral nerves to the central nervous system. The spine consists of 33 vertebral segments, the upper 24 are articulating and separated by each other by intervertebral discs, and the lower nine are fused in adults. Each disc acts as a cushion-like joint between vertebral segments, allowing for slight movement while holding the segments together and acting as a shock-absorber. In this section the effects of vibration on the spine and associated structures will be explored.

The vertebral segments of the spine display the same effect from vibration as any other bone, in that there is an increase in BMD after vibration stimulation [23]. What is of great interest however is the effects of vibration on the intervertebral discs (IVD), as the degeneration of the intervertebral discs is considered as an important pathogeny for spinal disorders and low back pain [229]. Desmoulin et al. (2013) tested bovine IVD in multiples studies with 0–200 Hz vibration and found an increase in mRNA expression of aggrecan, collagen type I and II, decorin, and versican in significant amounts [230,231,232]. Another study found similar results in living mice treated with 15–90 Hz vibration at 0.3 g, showing an increase in the expression of aggrecan, biglycan, decorin, type I collagen, and Sox9 [233]. Liang et al. (2017) found that vertical and whole body vibration in rats (0.49 g at 30 Hz) increased expression of aggrecan, collagen type I and II, and decorin [234]. Aggrecan is a proteoglycan with primarily a mechanical function in the tissue matrix and is vital for disc health. Analysis of excised painful IVD has shown the presence of nerves and blood vessels that have intruded into the disc, and this has been suggested to be a result of altered aggrecan content in the IVD [235]. Decorin is a small proteoglycan produced by neurons and astrocytes and has an anti-inflammatory effect and regulates scarring in the spinal cord. For this reason, Decorin has been suggested as a treatment for spinal cord injury [236]. Biglycan is a small molecule that is important in the mineralization of bone and also plays a role in the osmotic swelling in the IVD, and versican is a large proteoglycan, which functions in cell adhesion and cell signaling [234]. Collagen type I and II are important components of the annulus fibrosus and nucleus pulposus respectively [234]. Taken together, the expression of these genes produces an anabolic effect, which improves the health of the discs and maintains its hydration and shock absorbing capabilities. In low back pain, abnormal mechanical loading leads to internal disc disruption [237,238,239], cell-mediated loss of water content and disc height, and is associated with a loss of aggrecan and collagen content within the disc [240,241]. The disc releases proinflammatory cytokines promoting the infiltration and activation of immune cells that potentiate nerve and blood vessel growth further. The degenerated disc and the immune cells begin to produce neurogenic factors that activate nociceptive nerve signals to produce the sensation of disc related pain [242]. Therefore, vibration treatment may serve to counteract these types of effects seen in the progression of disc degeneration that leads to pain.

#### Application: Focused Vibration for Chronic Back Pain

An important emerging application for vibration among human patients is for the treatment of spine related chronic back and neck pain. Desmoulin et al. (2007 and 2007) conducted two similar studies recruiting both chronic neck pain and chronic back pain patients and tested vibration (80–120 Hz) delivered directly to the C1 vertebrae of the spine. They found a significant improvement in pain, an improvement in range of motion, and a reduction in pain medication dosage after one month of vibration stimulation. The improved pain may be due to the anabolic effects of vibration on the intervertebral discs, which could have an anti-inflammatory effect to prevent discogenic back pain. Another possible mechanism may be that vibration analgesia is produced by neural input at specific frequencies that alter central nervous system processes [107,243,244], however it is still unclear how this may work. Range of motion of the spine has also improved and is likely due to vibration. Keller et al. (2000) quantified the mobility characteristics of human thoracolumbar spines with transient vibration stimulation [245]. They found that the thoracic region of the spine is more mobile than the lumbar spine, and found 40 Hz and 80 Hz to be the best for inducing mobility. Although the mechanism is not yet fully clear, it is suggested that vibrations at the resonant frequency of the spine are the most potent at inducing improvements in mobility. These findings support the results of another clinical study by Desmoulin et al. (2012) that found that focal vibration stimulation of the C1 of the vertebrae translated to postural improvements of spinal alignment in the cervical spine [147]. This study measured the mean axis of rotation of cervical spines from flexion–extension X-ray imaging to quantify the degree of spinal alignment. Vibration delivered to the cervical spine was able to significantly correct misaligned cervical spines of 44 patients with chronic neck pain. Since spinal alignment is an important factor in chiropractic and orthopedic practice and its role in back pain is established [246], the correction of spinal posture using vibration may be another mechanism behind pain relief.

## 5. Discussion and Conclusions

### 5.1. Reporting Suggestions

An important observation from this review of vibration literature is how precisely the application of vibration is reported in studies. Research studies typically report some combination of the following: (1) the frequency of the vibratory stimulant in Hertz, (2) the amplitude in mm of the vibration, which is defined as the maximum displacement by a point on a vibrating body from its equilibrium position, (3) the acceleration of a point on the vibrating body measured in metric units (m/s^2^) or units of gravitational constant g, where 1 g = 9.81 m/s^2^, (4) the pressure of vibration measured as dyn/cm^2^, which indicates the force of 1 g accelerated by 1 cm per second squared; or as pGz indicating periodic (*p*) vibrational acceleration (G) along the z axis (horizontal), which in research is typically between 1 and 4 Hz, and (5) the duration of the stimulant as well as how often it is applied over days/weeks/months.

There are two main inconsistencies regarding reporting the characteristics of vibrations. First, the inconsistency of what is considered “high frequency” and what is considered “low frequency”. For example, some studies describe vibrations of 30–100 Hz as high frequency [206,208,220,222] (the perspective of a whole body platform that moves mechanically), whereas other studies report anywhere from 0 to 200 Hz as low frequency [70,115,119,155,180] (the perspective of sound). This is largely due to the scientific contexts in which researchers are interested, but such contextual labels make the synthesis of knowledge in vibration more difficult than it needs to be. We recommend simply saying “vibration at X frequency”. If one wishes to be descriptive, it is better to describe the nature of the vibration or applied area rather than saying it is high or low. For example, one may investigate the effects of percussive vibration, acoustic vibration, globally, or focally applied vibration. We used the term “low frequency” to describe 0–250 Hz vibration because this paper is presented in the context of music and sound.

The second inconsistency is in reporting of the amount of surface of the body that is stimulated and the relative distribution of the vibratory effect. For studies in WBV the entire body is stimulated but the actual vibratory transmission can be dependent on posture and stance. With vibroacoustic stimulation devices, the application can be almost the entire body with speakers above the knee, lower back, upper back, and neck [40] or primarily the back with a single transducer in the centre of the back [41], or only hands and feet with small piezoelectric devices [247], or as small as a pencil-like probe [32,147]. Therefore, we recommend to add a sixth reporting category: (6) the region of the body that is directly stimulated by vibration (e.g., spine, low back, neck, feet, and whole-body).

As a result of the different methods of measurement, an important consideration is which measurements should be reported at a minimum to replicate studies of vibration on human health and accurately compare different studies. Not all six categories of measurement described above need to be reported because some allow conversion to other units. For example, displacement can be determined by velocity and frequency, velocity can be determined by frequency and displacement, acceleration can be determined by frequency and displacement, and pressure can be determined by force (a function of acceleration and mass) and the area applied. Therefore, reporting standards for vibration studies on health should have a pragmatic number of measurements that can allow conversions to other units if need be. We recommend a minimum of (4) measurements: the frequency (in Hz), the acceleration (in g), the duration of stimulus (time applied and over how many days), and the area of the body stimulated (e.g., spine, back, whole-body, or a measured area in units). The area of applied vibration is a new but important reporting standard to consider.

### 5.2. Clinical Implications

This attempt to “map the landscape” of the mechanisms of vibrational effect on the body has shown that research is beginning to identify how vibration affects “blood, brain, and bone” but that the means of the effect is highly complex. There is ample evidence that whether a person is standing in front of a tower of speakers at a rock concert, dancing to a pulsating beat, feeling the quake of pipe organ pedal tone, travelling on the underground train, riding a carriage on cobble stones, using a handheld vibrator, standing on an oscillating platform in a gym, sitting in a vibroacoustic chair, or having the spine treated with a vibrating stylus there is potential for effects that can affect health positively or negatively.

Throughout the paper we pointed to potential clinical applications. In some cases, these are already being used as in the case of whole body vibration for osteoporosis, or focal application to the spine for orthopedic conditions. However, most of the potential applications still need to be clinically recognized and commercialized in medically regulated products so that specific claims and, consequently, medical prescription can become generally practiced. The potential for this is indicated by the research in haemodynamics, in neurology, and in musculoskeletal conditions.

Professional organizations to promote and research these effects of vibration include VIBRAC (https://www.vibrac.fi/) (accessed on 21 April 2021), which is focused on vibracoustic therapy for a large but mainly unspecified range of health conditions and WAVEX: World Association of Vibration Exercise Experts (https://internationalwavexmeeting. wordpress.com/) (accessed on 21 April 2021) focused on WBV for physical and cognitive health.

Although mechanisms of action stemming from vibration appear to be clinically relevant in many situations, caution must be urged that in many cases the research employed animal studies. In many cases results from animal studies can be replicated in humans but that is not a certainty. Consequently, translation to clinical applications is not immediate and may not be successful.

### 5.3. Future Research

Our review has shown that much more specific research is needed. For example, research needs to determine whether vibroacoustic stimulation of the torso in a chair has similar effects as whole body vibration on a platform on the muscles in frail people. Research needs to explore more fully the effect of frequency on specific mechanisms. For example, how is nitric oxide positively correlated and prostaglandin E_2_ negatively correlated with frequency and, therefore, what is a target frequency for what specific effect. Similarly, our review showed examples of how vibration at one amplitude was positive and at another was negative. The complexity of mechanism response to vibration makes clinical application and conclusion of effect difficult. Interacting variables on multiple mechanisms including duration, strength, cycle, frequency, application location and area, and patient group make control of specific outcomes a challenge and frame the agenda for urgently needed research to advance this potentially potent form of treatment.

An important observation from this effort to “map the landscape” is that there is not an integrated “field” of vibrational research. Rather there are individual silos that seem to function in isolation of each other. This even applies to the type of stimulus. WBV has developed as one silo because of the device and strong application within athletics and sports training and medicine. Vibroacoustic therapy is another silo, less developed although not new, and with less awareness of mechanism in its research. The most diverse and so not even qualifying as a “silo” is focal vibration with applications ranging from orthopaedic treatment, to temporomandibular joint pain management, to orthodontic tooth movement, to tissue engineering, or to orthopedics. The reality may be that the person in the gym seeking to strengthen the muscles of the body core on a vibrating platform may be doing much more than they realize. It is time for these separate but related research streams to explore commonalities and shared research questions to move this clearly important field forward to advance clinical applications in healthcare.

## Data Availability

Not applicable.
